# Use of an Ophthalmic Viscosurgical Device for Experimental Retinal Detachment in Rabbit Eyes

**DOI:** 10.3390/jfb4010006

**Published:** 2013-01-18

**Authors:** Akira Hirata, Soichiro Yamamoto, Satoshi Okinami

**Affiliations:** Department of Ophthalmology, Faculty of Medicine, Saga University, 5-1-1, Nabeshima, Saga 849-8501, Japan; E-Mails: stentorviolin@gmail.com (S.Y.); okinami@cc.saga-u.ac.jp (S.O.)

**Keywords:** OVD, optical coherence tomography, retinal adhesive, retinal detachment, retinal glue, vitrectomy

## Abstract

To investigate the temporary tamponade effects of an ophthalmic viscosurgical device (OVD) for experimental retinal tears, we performed vitrectomy in four rabbit eyes and created a posterior vitreous detachment and artificial retinal tear to produce retinal detachment. The retina was flattened with liquid perfluorocarbon (PFC), the area peripheral to the tear was photocoagulated, an OVD was applied to the retinal tear surface below the PFC and the PFC was removed by aspiration. In the control group, PFC was removed without application of OVD. At one, three and seven days postoperatively, funduscopy and optical coherence tomography (OCT) were performed to examine the sealing process of the retinal tear. In OVD-treated eyes, the OVD remained on the retinal surface, and the retinal tear was patched for ≥ 3 days postoperatively. By seven days postoperatively, the OVD on the retinal surface had disappeared, and the retina was reattached. In control eyes, the edge of the retinal tear was rolled, and retinal detachment persisted. In OVD-treated eyes, the border of the retinal tear was indistinct, and the defect area was significantly decreased. These results show that application of an OVD effectively seals retinal tears and eliminates retinal detachments.

## 1. Introduction

Vitreous surgery is commonly performed for rhegmatogenous retinal detachments [1]. The basic procedure is vitreous dissection around the retinal tear, repositioning of the retina by fluid-gas exchange, laser photocoagulation around the tear and a certain period of gas tamponade after surgery [2,3,4,5]. The introduction of minimally invasive (small-gauge) vitreous surgery has shortened operative times and reduced postoperative inflammation, but resting in a prone position after surgery is still necessary, and stress to the patient is significant. To reduce postsurgical prone positioning, sealing of the retinal tear when surgery is completed is ideal. Various bioadhesives, such as fibrin glue [6], cyanoacrylate [7], hydrogel compound [8], mussel adhesive protein [9], transforming growth factor [10] and bioresorbable membrane [11] have been proposed as retinal adhesives, but none have been effectively used to date.

In the treatment of rhegmatogenous retinal detachment, the tamponade effect of gas injected into the vitreous cavity lasts from only a few days to a couple of weeks, depending on the location of the retinal tears. Therefore, in some cases, the restoration of retinal pigment epithelium (RPE) pump action, retinal-RPE structural restoration and sealing of the retinal tear by scar formation at retinal photocoagulation sites are not supported sufficiently. In experimental retinal detachment, RPE pump action is restored within 24 h, and a few weeks are required for subsequent retina-RPE structural restoration and scar formation at retinal photocoagulation sites. Therefore, by temporarily patching the retinal tear rather than creating gas tamponade, subsequent sealing of the retinal tear without tamponade and its associated discomfort can hopefully be promoted.

Ophthalmic viscosurgical devices (OVDs) are widely used in anterior eye procedures, such as cataract surgery, and their safety has been established [12]. OVDs that contain chondroitin sulfate are called dispersive OVDs and provide excellent corneal endothelial protection, because of good retention ability [13,14]. In posterior eye surgery, application of vitreous substitutes mainly using hyaluronan formulations for temporary tamponade has been attempted, but none have yet been established [15].

This study investigated the adhesive and retention abilities of a dispersive OVD on the retina, including its effects on sealing of retinal tears. An intentional retinal tear and localized retinal detachment were created in rabbit eyes, and then, after flattening of the retina with liquid perfluorocarbon (PFC), retinal photocoagulation was performed and the OVD was applied on the retina beneath the PFC. Next, the vitreous cavity was filled with intraocular irrigating solution, without fluid-gas exchange, and surgery was completed. Postoperative examination was performed using optical coherence tomography (OCT), and the status of the tear was examined under scanning electron microscopy (SEM).

## 2. Results and Discussion

### 2.1. Effects of Dispersive OVD on Experimental Retinal Detachment Examined by OCT

**Figure 1:**
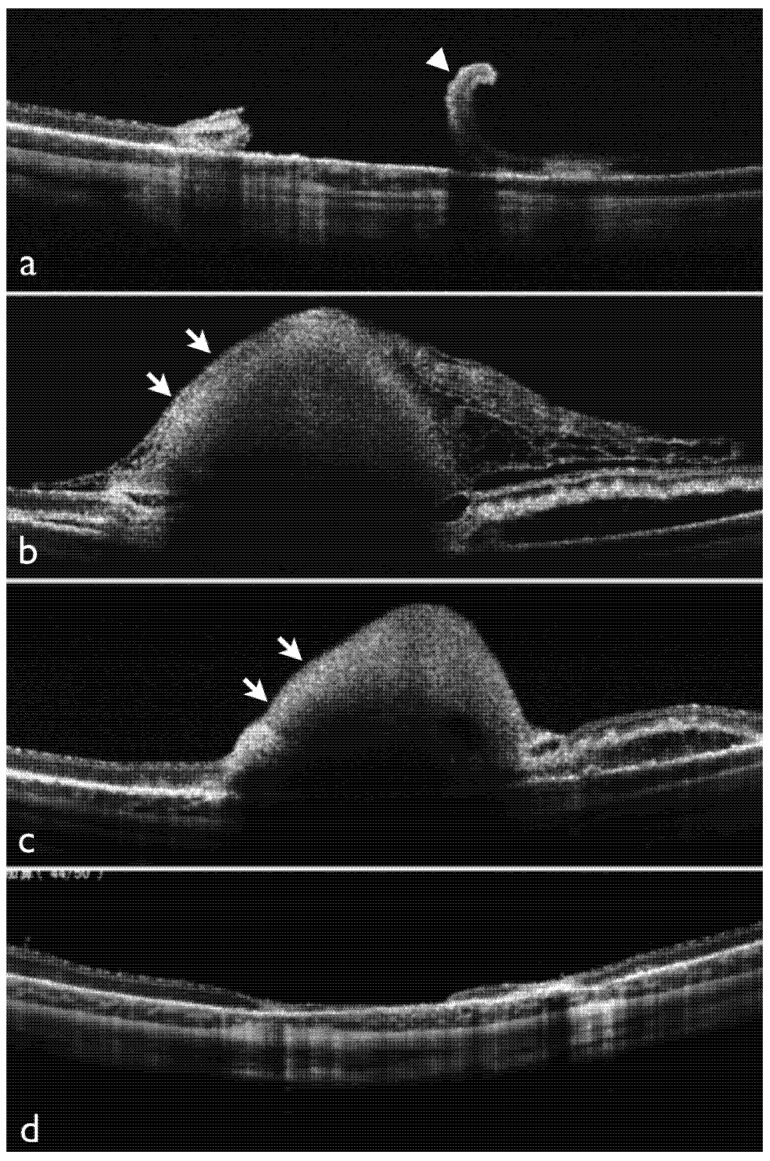
Optical coherence tomography (OCT) findings after vitrectomy with application of dispersive ophthalmic viscosurgical device (OVD). (**a**) OCT findings on day 7 in the control group. The retinal tear is open and curled, and retinal detachment persists; (**b**) OCT findings after 1 day in an eye with surgery using dispersive OVD (arrows) (dispersive OVD group). Subretinal fluid is still present, but the OVD has completely patched the tear; (**c**) OCT findings after 3 days. The dispersive OVD has slightly decreased, but the amount is still sufficient to patch the retinal tear; (**d**) OCT findings after 7 days. The OVD has disappeared, the tear site has been repositioned and retinal detachment is no longer present.

Figure 1 shows OCT findings in rabbit eyes after vitrectomy. In control eyes, in which surgery was completed without application of the dispersive OVD, the retina was repositioned with purified perfluoro-n-octane liquid, and photocoagulation was performed. The purified perfluoro-n-octane liquid was then removed, and surgery was completed. On the day after surgery, OCT revealed slight retinal detachment, and the retinal tear was open. Even on postoperative day 7, although no progression was seen, the retinal detachment near the tear still persisted (Figure 1a).

Postoperative inflammation in subjects receiving patching of the retinal tear with a dispersive OVD intraoperatively did not differ from that in the control group; there were no apparent inflammatory cells in the anterior chamber, fibrin formation in the vitreous cavity or retinal edema. In addition, intraocular pressure was not increased. In all eyes on the day after surgery, OCT clearly depicted patching of the retinal tear by the dispersive OVD (Figure 1b). On postoperative day 3, OCT showed that although the amount of dispersive OVD was slightly decreased, the retinal tear remained adequately patched (Figure 1c). On postoperative day 7, OCT showed that the dispersive OVD had disappeared in all cases. The retinal tear site was completely repositioned and no persistent retinal detachment was observed (Figure 1d).

### 2.2. Observation of the Retinal Surface by SEM

**Figure 2:**
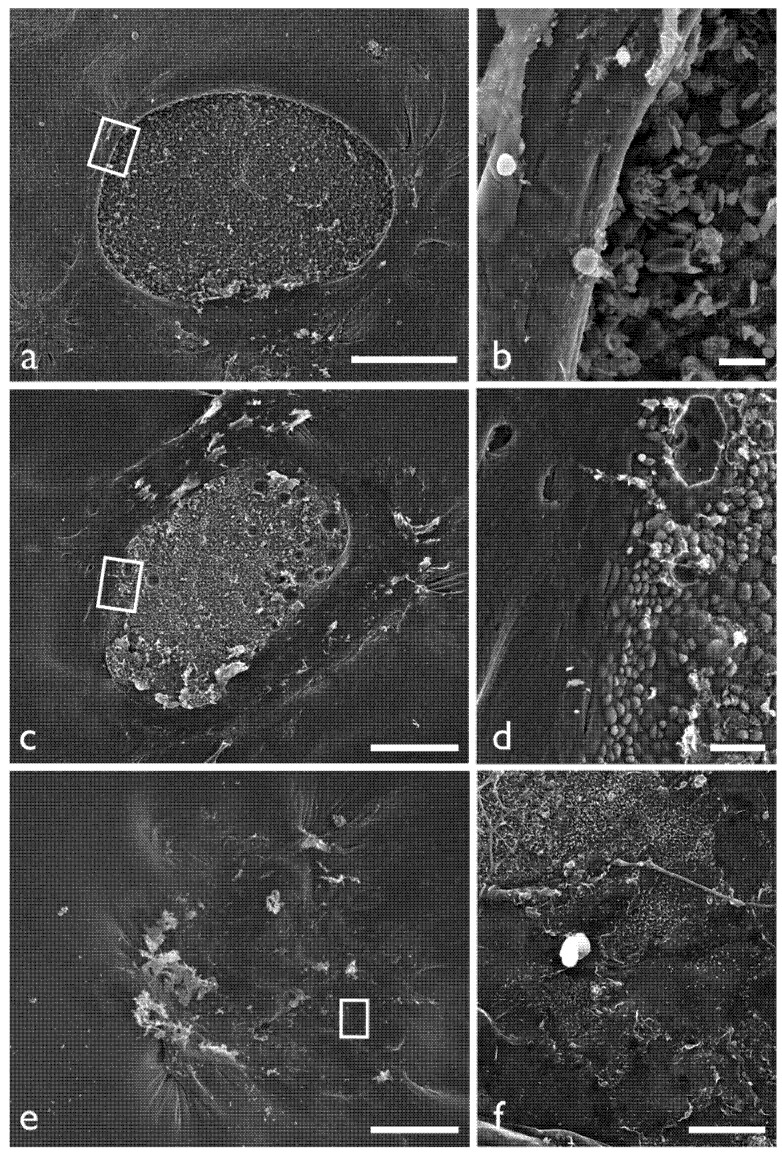
Scanning electron microscopy (SEM) findings of retinal surface in control eye and eyes with application of dispersive OVD. (**a,b**) SEM findings in a control eye. The retinal tear is clearly visible. A magnified image of the area within the square (**b**) shows some floating of the tear edge; (**c,d**) SEM findings in an eye with dispersive OVD. The exposed area of retinal pigment epithelium (RPE) inside the retinal tear is clearly apparent; but a magnified image of the area within the square (**d**) shows an indistinct border between the retinal tear edge and RPE; (**e,f**) SEM findings in another eye with dispersive OVD. The area of exposed RPE is indistinct; A magnified image (**f**) shows the site of the retinal tear irregularly covered by flat epithelial cells. Bars: 50 μm for (**a**), (**c**) and (**e**); 50 µm for (**b**) and (**d**); and 20 µm for (**f**).

The retinal surface was examined by SEM in the control and dispersive OVD groups (Figure 2). In the control group, the border of the retinal tear was clearly visible, and on a highly magnified view of the tear edge, the retina near the tear was floating. However, in the dispersive OVD group, the border of the retinal tear was indistinct, and the area of exposed RPE was reduced. In another case, the tear was almost indistinct, and the exposed area was covered with glial cells. The area of exposed RPE was measured at 1.46 ± 0.44 mm^2^ in the control group and 0.45 ± 0.53 mm^2^ in the dispersive OVD group. The area of exposed RPE was significantly lower in the dispersive OVD group (*p* = 0.029, Mann-Whitney test).

### 2.3. Discussion

Dispersive OVDs are currently widely used in cataract surgery, and compared to OVDs with hyaluronan alone (cohesive OVDs), they have stronger adhesive ability [12,16]. The present study was conducted based on the premise that this strong adhesive ability would also be advantageous in the sealing process of retinal tears. We examined the effectiveness of retinal repositioning by short-term patching of a retinal tear with a dispersive OVD in four rabbit eyes. No postoperative inflammation was seen with use of a dispersive OVD, and all rabbit eyes treated with an OVD achieved patching of the retinal tear for ≥3 days and, ultimately, repositioning of the retina.

Vitrectomy is now widely performed for retinal detachment, and the frequency of vitrectomy for treatment of rhegmatogenous retinal detachment is expected to increase further [1]. However, gas tamponade is necessary with current surgical techniques, requiring patients to maintain a prone position. This study found that a dispersive OVD provided patching, albeit temporarily, of retinal tears. This may be promising to reduce the physical and psychological discomfort associated with conventional gas tamponade.

Another interesting finding was that in dispersive OVD-treated eyes, not only was the retinal tear repositioned, but also the size of the tear was significantly decreased. Patching of retinal tears with an OVD prevents contact between vitreous fluid and retinal tear surface cells, which may promote the growth of glial cells. Hyaluronan, an OVD component, is also known to regulate cell proliferation [17,18,19]. The significance of OVDs as extracellular signaling molecules warrants further investigation.

In this study, the retina was repositioned with purified perfluoro-n-octane liquid before injecting the OVD. Injection of an OVD between the perfluoro-n-octane liquid and retinal surface can prevent reduced adhesion due to excessive moisture. In addition, pressure bonding to the retinal surface occurs due to the high specific gravity of perfluoro-n-octane liquid.

## 3. Experimental Section

### 3.1. Animals

This study was performed using eight 12-week old Dutch rabbits weighing 2.0–2.5 kg (Biotek, Saga, Japan). The study was conducted in accordance with the Statement for the Use of Animals in Ophthalmic and Vision Research and the guidelines of the Committee on Animal Research of Saga University.

### 3.2. Vitrectomy with Experimental Retinal Detachment

**Figure 3:**
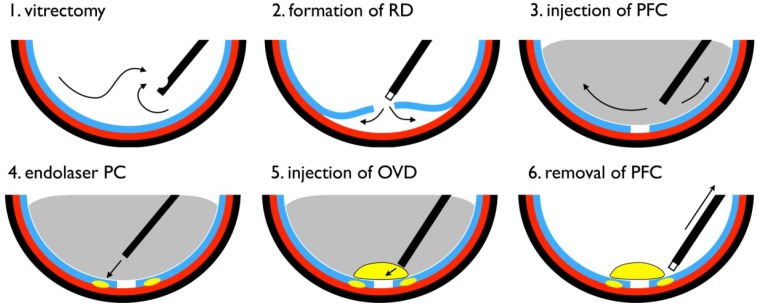
Diagram of surgical procedure. RD, retinal detachment; PFC, perfluoro-n-octane liquid; PC, photocoagulation; OVD, ophthalmic viscosurgical device.

**Figure 4:**
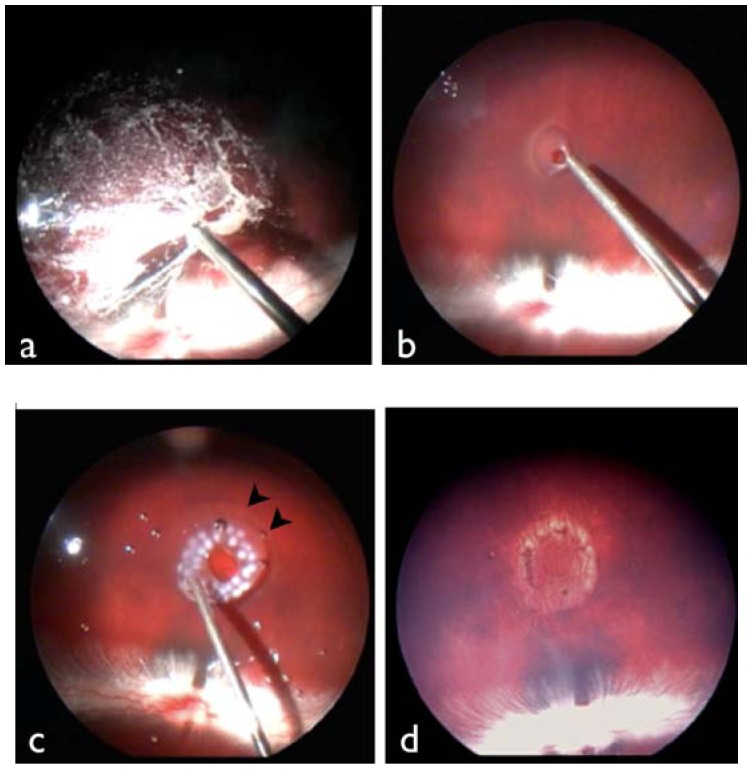
Fundus findings during and 7 days after surgery. (**a**) Core vitrectomy with aid of triamcinolone acetonide; (**b**) Creation of retinal tear inferior to the optic disc using an extrusion needle; (**c**) Injection of purified perfluoro-n-octane liquid, photocoagulation of the area around the tear and injection of dispersive OVD onto the retinal surface; (**d**) After 7 days, sealing of the retinal tear and scarring of the photocoagulation sites is apparent.

Vitrectomy was performed as described in a previous report [20]. In brief, after adequate dilation of rabbit eyes using a mixture of 0.5% tropicamide and 0.5% phenylephrine hydrochloride, rabbits were anesthetized with pentobarbital and xylazine. At 1 mm posterior to the temporal inferior sclerocorneal limbus, a trocar was placed as an infusion port using a 23-gauge infusion cannula with a sharp solid trocar blade (Accurus Surgical System 23-gauge; Alcon Japan, Tokyo, Japan). The cannula was connected to a reservoir of balanced salt solution (BSS; Alcon Japan, Tokyo, Japan) and inserted on the trocar. Trocars were similarly placed temporo-superiorly and naso-superiorly for insertion of a vitreous cutter and light pipe. Core vitrectomy was performed, followed by creation of a posterior vitreous detachment using triamcinolone acetonide (Figure 3, Figure 4a). After adequate vitreous dissection, a one-half to one-third disk diameter (DD) retinal tear was created using an extrusion needle at a location 2 DD inferior to the optic disc (Figure 3, Figure 4b). Irrigating fluid was injected into the retinal tear to create a localized retinal detachment (diameter: 2 DD). Next, purified perfluoro-n-octane liquid (Perfluoron; Alcon Japan) was injected into the vitreous cavity, the detached retina was temporarily flattened, and laser photocoagulation around the retinal tear was performed. Ten microliters of a dispersive OVD (Viscoat; Alcon Japan) was then injected on the retinal surface beneath the perfluoro-n-octane liquid to patch the retinal tear, and after 5 min, the purified perfluoro-n-octane liquid was aspirated and removed (Figure 3, Figure 4c). All trocar insertion sites were closed with 8-0 Vicryl, and antibiotic eye drops were instilled. The retinal tear was examined postoperatively on days 1, 3, 5 and 7 by funduscopy (Figure 4d) and spectral domain OCT (RS-3000; Nidek, Gamagori, Japan).

### 3.3. Scanning Electron Microscopy

Examination of the retinal surface by SEM was performed to assess morphological changes in the retinal tear after vitrectomy with and without application of the dispersive OVD. Immediately after rabbits were sacrificed with an overdose of intravenously injected pentobarbital, the eyeball was enucleated, a mixture of 2.5% glutaraldehyde and 2% paraformaldehyde in 0.1 M phosphate buffer was injected into the eye, and 15 min later, the eye was cut at the equator to make posterior cups. After postfixation with 2% tannic acid stain and osmium tetroxide, dehydration through an alcohol series and freeze-drying with t-butanol, the specimen was coated with platinum and examined by SEM (JSM-5200LV; JEOL, Tokyo, Japan). The area of the retinal tear (area of exposed RPE) was measured from the photographic images and the dispersive OVD and control groups were compared. Results were analyzed using the Mann-Whitney test, with a statistical significance level of *p* < 0.05.

## 4. Conclusions

In conclusion, although conducted in only a small number of rabbit eyes, our results suggest that this may offer a novel procedure in surgery for retinal detachment. Although many factors must be taken into consideration, including size of the retinal tear, extent of detachment and the type and amount of OVD, Vitrectomy using an OVD for retinal detachment repair may be promising to reduce patient discomfort in the early postoperative period and to achieve an early return to society.
